# Soil Respiration Dynamics and Environmental Controls Across Montane Forests of Nepal

**DOI:** 10.1002/pei3.70159

**Published:** 2026-05-11

**Authors:** Sanu Raja Maharjan, Deepa Dhital, Lal Bahadur Thapa, Ram Kailash Prasad Yadav, Chandra Prasad Pokhrel

**Affiliations:** ^1^ Central Department of Botany Institute of Science and Technology, Tribhuvan University Kathmandu Nepal; ^2^ Department of Botany, Trichandra Multiple Campus Tribhuvan University Kathmandu Nepal; ^3^ Faculty of Science Nepal Academy of Science and Technology (NAST) Lalitpur Nepal

**Keywords:** closed chamber method, soil carbon emission, soil moisture, soil temperature, temperature sensitivity

## Abstract

Soil respiration (*R*_S_) represents a major process of release of carbon dioxide (CO_2_) from soil to atmospheric carbon pools. Measurements of soil respiration help to understand the dynamics of carbon in ecosystems. This study examines the soil respiration rate and the effect of environmental variables in different forests along elevation gradient. This study was conducted in three distinct montane forest types distributed along an elevational gradient in the middle mountain region of Nepal, namely *Schima*‐*Castanopsis* Forest, Oak Laurel Forest, and Evergreen Oak Forest. In each forest type, 10 circular chambers were installed for measuring soil respiration. Soil CO_2_ efflux was measured monthly for 1 year, using the “closed chamber method” with an infrared gas analyzer. Soil respiration rate was modeled as a function of soil temperature and moisture using a generalized linear model (GLM). Soil respiration rate varied significantly among the forest types, ranging from 274.7 to 352.4 mg CO_2_ m^−2^ h^−1^ and demonstrated seasonal changes with a summer peak. Soil respiration was significantly higher in Evergreen Oak Forest than Oak Laurel Forest and *Schima*‐*Castanopsis* Forest. Response of soil respiration to soil temperature and soil water content indicated a significant exponential relationship in all the forests. Soil respiration showed a strong correlation with soil temperature than soil water content. Temperature sensitivity (Q10) of soil respiration was higher in the forest of upper elevation (Evergreen Oak Forest, Q10 = 3.9) than in the lower elevation forests (Oak Laurel Forest, Q10 = 2.7; *Schima Castanopsis* Forest, Q10 = 2.5), indicating that soil respiration in the Evergreen Oak Forest is more responsive to temperature changes. Hence, forests at higher elevation are highly susceptible in the context of future climate warming due to enhanced efflux of soil CO_2_. This study highlights the necessity of incorporating belowground carbon processes into climate policy and sustainable forest management frameworks in Nepal.

## 1 Introduction

Forest sequesters carbon dioxide (CO_2_) from the atmosphere and stores it in the form of biomass and soil. Based on the ground based measurements and earth observation data, globally forests contribute to net carbon sequestration of 7.6 ± 49 Gt CO_2_ year^−1^ with nearly double the sequestration than emission through respiration (Harris et al. 2021). Hence, a significant portion of sequestered carbon dioxide, roughly half of the CO_2_ absorbed, is released back to the atmosphere through respiration by plants and organisms (Gonzalez‐Meler et al. 2004).

Soil respiration (*R*_S_) is a major process of CO_2_ efflux in terrestrial ecosystems and acts as a dominant process in the overall ecosystem carbon balance (Tan et al. 2013). The release of CO_2_ during soil respiration results due to two combined processes: respiration by soil microorganisms within the soil and plant roots (Schlesinger and Andrews 2000). Soil respiration process has important ecological implications and acts as a key indicator of overall soil health and microbial activity affecting ecosystem productivity (Goupil and Nkongolo 2014). On the other hand, soil respiration has significance as it returns a huge amount of CO_2_ and contributes to the global carbon cycle (Schlesinger and Andrews 2000).

Globally, a significant amount of CO_2_ is released to the atmosphere by the soil respiration process, often exceeding emissions from the combustion of fossil fuels (Bond‐Lamberty and Thomson 2010). As a major pathway for CO_2_ release from soil to atmospheric carbon pools, soil respiration plays a crucial role in climate warming. Conversely, global warming may enhance soil respiration, further impacting atmospheric CO_2_ concentrations (Davidson et al. 2006). Carbon emission from forest soil is influenced by anthropogenic disturbances and forest management activities which can alter the physical and biological environment of soil. Increased forest disturbances such as logging, lopping, fire, etc. can bring change in organic matter which can alter microbial activities and decomposition rate (Concilio et al. 2006). Therefore, understanding the process and status of CO_2_ efflux from soil is essential for evaluating carbon dynamics in forest ecosystems.

Recent research trends emphasize understanding of soil respiration in different ecosystems. Soil respiration is a complex process regulated by multiple factors such as vegetation type, soil temperature, moisture content, and plant productivity (Chen et al. 2024; Raich and Tufekciogul 2000; Zhang et al. 2021). Extensive studies exist on the estimation of soil respiration and its relationship with environmental factors (Zhu et al. 2013). Soil temperature and moisture are often the most influential factors regulating soil respiration rate, which affects the activity of both roots and microbial organisms (Hanson et al. 2000). Higher global temperatures due to climate change result in an increase in soil respiration rates, releasing more CO_2_ into the atmosphere. This creates an imbalance in the global carbon cycle, further amplifying global warming.

Vegetation type may also affect soil respiration by influencing root respiration, organic matter supply, and soil microclimate (Raich and Tufekciogul 2000). Thus, analyzing the effect of environmental factors on soil respiration is essential to improve our knowledge on global carbon cycle and model prediction of soil respiration under various environmental conditions (Hopkins et al. 2013).

The elevation gradient in mountains reveals that, due to climatic variations, it supports different forest types, even within similar geographical regions (Malhi et al. 2010). Hence, elevation gradients have been widely used to assess variations in vegetation type and soil carbon stock and flows in response to temperature. Studies suggested that the soil carbon stock in forest increases with elevation, while the soil respiration rate decreases due to lower temperatures and reduced plant biomass (Takeda et al. 2023). There is a consensus that the sensitivity of soil respiration to temperature (Q10) increases with elevation (Okello et al. 2023), indicating that proportionately higher carbon emissions can be predicted from soils at higher elevations.

The respiration rate also varied significantly depending on tree species composition (Kumar et al. 2023). Additionally, in mountainous regions, soil respiration may be influenced by complex topographical factors such as slope, orientation, and geological substrates (Badraghi et al. 2021). Soil respiration rates vary among different forests due to large variations in spatial and temporal factors. Moreover, soil respiration varied seasonally within the same forest type with soil moisture and temperature change as key environmental determinants (Maharjan et al. 2025). On the other hand, soil respiration rate differs among forests due to variation in organic input from leaf litter and the difference in soil characteristics (Epron et al. 2004).

Elevation gradients provide a natural framework for climate change studies. There is limited research on soil respiration in Himalayan forests, and the mechanisms regulating this process, as well as their responses to changing environmental conditions, remain poorly understood. Hence, studies are necessary to understand how soil carbon fluxes of forest ecosystems respond to ongoing and future warming. This study would provide baseline data on soil carbon emission of forest ecosystems, which would be helpful in forest management planning and predicting soil respiration under future climate change scenarios. We hypothesize that the soil respiration rate varies significantly across different forest types along elevation gradient due to differences in environmental factors and vegetation composition. This study aims to: (i) examine seasonal variations in soil temperature and soil water content across different forest types, (ii) assess soil respiration rate in forests along an altitudinal gradient, and (iii) analyze the influence of key environmental factors, particularly soil temperature, and moisture, on soil respiration rates.

### 1.1 Site Description

**FIGURE 1:**
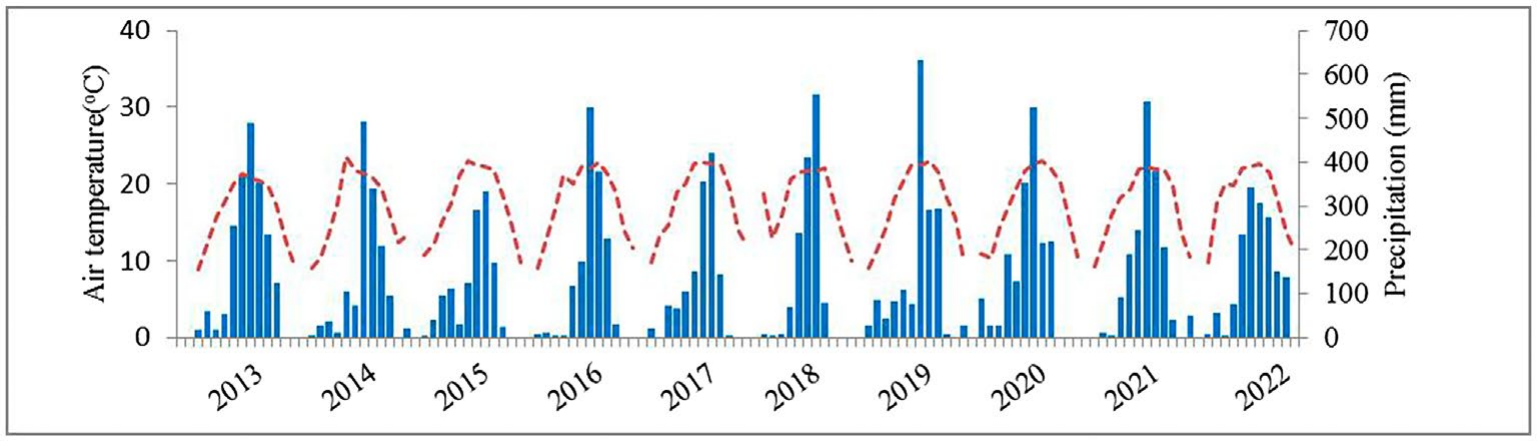
Mean monthly precipitation and average air temperature of study area (Godawari weather station, 2013–2022). *Data source:* Department of hydrology and meteorology, Government of Nepal.

The study was conducted in three different forest types of Phulchoki hill, Lalitpur district in the middle mountainous region of Nepal. The hill comprises an elevation range from 1500 to 2760 m above mean sea level. The study area has a warm temperate climate, with an average annual precipitation (10 years) of 1527 mm. Annual climatic pattern can be divided into four distinct seasons with hot and rainy summer (June–August), mild autumn (September–November), cold and dry winter (December–February), and moderate and dry spring (March–May). Average minimum and maximum temperature (10 years data 2013–2022) were 10.7°C and 21.3°C, respectively. Figure 1 represents monthly average temperature and rainfall pattern, in the study area during the year 2013–2022.

The soils of the study area range in texture from sandy loam to loam. The upper horizons are fertile and enriched with organic matter (humus). Overall, the soils are acidic, with pH values ranging from 4.5 to 6.5.

**FIGURE 2:**
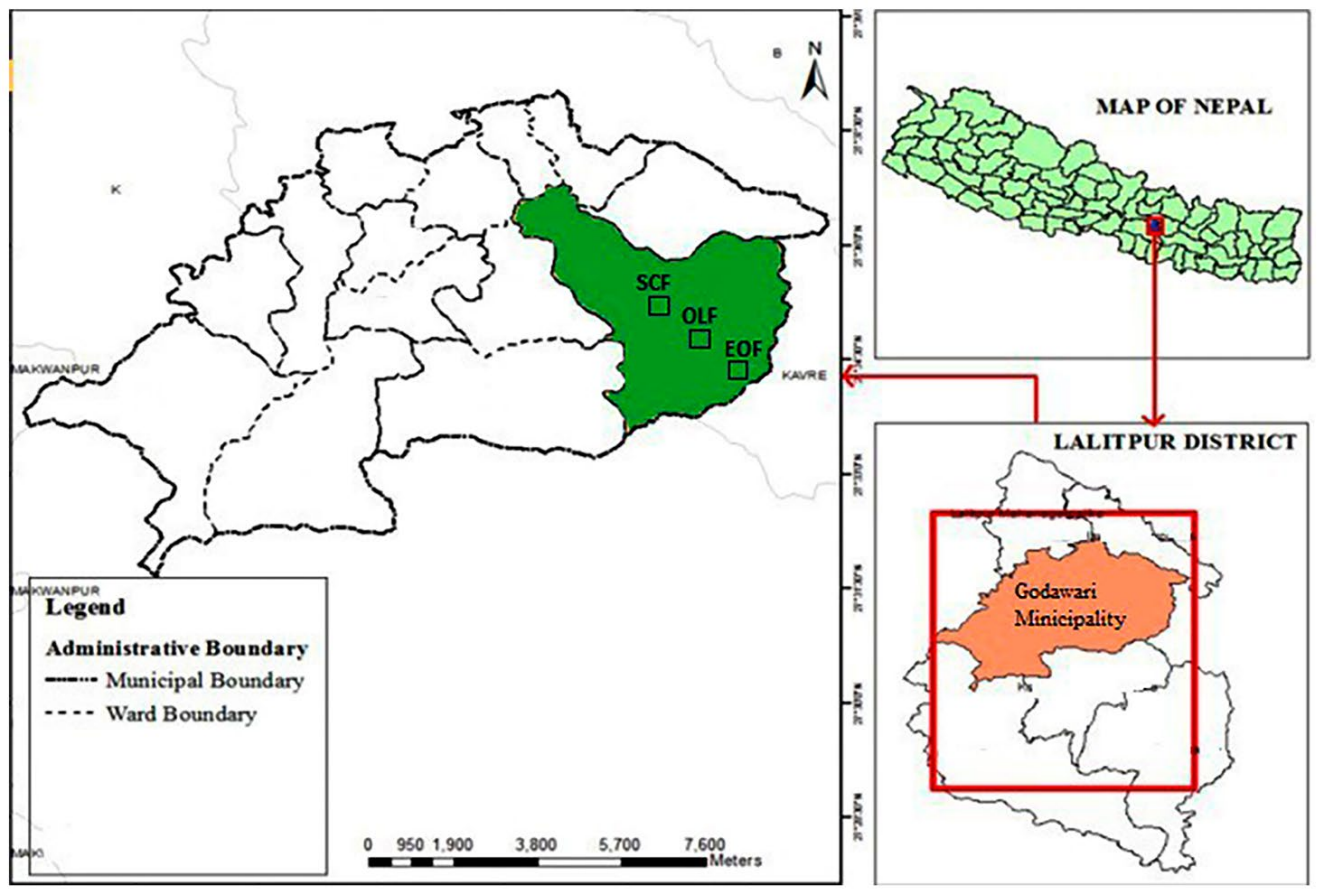
Map of study area showing study sites. EOF: Evergreen Oak Forest; OLF Oak‐Laurel Forest; SCF: *Schima‐Castanopsis* Forest.

The study was conducted in *Schima Castanopsis* Forest (1600 m), Oak‐Laurel Forest (2000 m), and Evergreen Oak Forest (2600 m) (Figure 2). The first site, *Schima‐Castanopsis* Forest (SCF), is characterized by a closed canopy with a higher density of small sized trees. The dominant tree species are *Schima wallichii* and *Castanopsis tribuloides*. The second site Oak Laurel forest (OLF) is dominated by *Quercus* spp. and *Myrica esculenta*. The third site, Evergreen Oak Forest (EOF) is located at a higher elevation and is dominated by mountain oak (*Q. semecarpifolia*). This forest features larger trees, but exhibits lower tree density. Additional forest characteristics are provided in Table 1.

**TABLE 1:** Site characteristics of the studied forest types.

Forest type	Altitude (m)	Basal area (m^2^ ha^−1^)	Density (stems ha^−1^)	Dominant trees
*Schima‐Castanopsis* Forest (SCF)	1600	28.5	793	*Schima wallichii, Castanopsis tribuloides, C* *. tribuloides* , *Carpinus viminea*
Oak Laurel Forest (OLF)	2100	35.4	433	*Quercus lanata, Quercus glauca, Myrica esculenta*
Evergreen Oak Forest (EOF)	2600	72.3	230	*Quercus semecarpifolia*

### 1.2 Determination of Soil Respiration Rate

An area of one hectare was selected in each forest type. Within the experimental area of each forest type, 10 circular chambers (collars) made of polyvinyl chloride (PVC), measuring 18 cm in diameter and 16 cm in height, were installed randomly with 2 cm of the base buried underground. All plants within the collar were removed, and the collars were left in place for the entire study period (from January to December 2022) to measure soil respiration rate.

Soil CO_2_ efflux was measured periodically within the chambers every month from January to December 2022, following the closed chamber method (Bekku et al. 1995). The method involved placing a closed chamber over the soil surface, equipped with a tightly fitted CO_2_ probe. An Infrared Gas Analyzer (IRGA) (Vaisala CARBOCAP CO_2_ probe GMP343) with GM 70 indicator was used to measure CO_2_ concentration within the chamber. The gas temperature and increase in CO_2_ concentration over time within the chamber was measured. Measurements were conducted in three continuous cycles at each collar, and the results were averaged to produce the mean *R*_S_ rate. All measurements were taken between 11:00 AM and 2:00 PM to avoid diurnal fluctuations in the rate. The soil carbon emission rate was calculated from the following equation (Bekku et al. 1995; Koizumi et al. 1999).

(1) $$R_{S}=\left(V/A\right)\times p\times \left(\Delta c/\Delta t\right)$$

where

- *R*_S_ = soil respiration (mg CO_2_ m^−2^ h^−1^)
- *V* = volume of air within the chamber (m^3^)
- *A* = area of the soil surface within the chamber (m^2^)
- *p* is the density of CO_2_ (mgm^−3^)
- Δ*c*/Δ*t* is the rate of change of the CO_2_ concentration in the air within the chamber (mg CO_2_ h^−1^).

The soil respiration measurement using the closed chamber method was ensured for accuracy through proper calibration, replication, and error assessment. The Infra‐red gas analyzer (IRGA) was calibrated using standard reference gas, to minimize instrument error. Leakage was checked to ensure the effectiveness of the chamber system. Measurements were taken over short durations to maintain linear CO_2_ accumulation.

Replication was carried out across multiple plots (10 plots for each forest type). Measurement was carried out in permanently installed collars to reduce disturbance, with 3 replicates per site (chamber), capturing spatial and temporal variability. Error margins were evaluated based on instrument precision, variability among replicates, and the linearity of CO_2_ concentration increase. Overall uncertainty was expressed using standard error, ensuring reliable estimates of soil respiration.

### 1.3 Annual Soil Respiration and Temperature Sensitivity (Q10 Value)

An exponential model was developed based on measured data to obtain the relationship between soil respiration and soil temperature in each forest.

(2) $$R_{S}T=a\times \exp^{b\times T}$$

where *R*_S_ *T* is soil respiration at a given temperature, *a* and *b* are constants, and *T* is temperature.

Hourly soil respiration rates of each forest type were estimated by substituting soil temperature data obtained from temperature data logger into the regression equation. The annual soil respiration of each forest type was obtained by summing the hourly soil respiration rates of the entire year. Temperature sensitivity of soil respiration (Q10 value) was calculated by inserting the value of *b* in equation

(3) $$Q10=\exp^{10\times b}$$

which describes the changes in soil respiration over a 10°C increase in soil temperature.

### 1.4 Measurement of Soil Temperature and Soil Water Content

Soil temperature was measured at 5 cm soil depth with the “Multi Stem Portable Thermometer” featuring an external sensing probe (AD‐5622, Japan). Similarly, measurement of soil water content was done by using a soil moisture meter, TRIME‐FM HD2 (Imko, Germany) at 5 cm depth near the chambers in each forest site. For continuous measurement of soil temperature throughout the measurement year, the “HOBO TidbiT v2 Temperature Data Logger” was installed within the soil in all three sites.

### 1.5 Data Analysis

Soil respiration was measured as the response variable, with forest type and season as categorical predictors, and soil moisture and temperature as continuous predictors. Normality was assessed with the Shapiro–Wilk test and differences across categorical predictors were tested using Analysis of Variance (ANOVA). Relationships between soil respiration and continuous predictors (soil temperature and moisture) were initially examined using linear regression. As the assumptions of normality and homoscedasticity were violated, gamma regression, a type of generalized linear model with a gamma distribution and log link was applied. Gamma regression was chosen to handle the non‐normality and heteroscedasticity of soil respiration data as it was positive, continuous, and right‐skewed. The log link function also captures the multiplicative effects of soil temperature and moisture, making it well‐suited for modeling ecological carbon fluxes. An exponential model was fitted as it best described the soil respiration response to soil moisture and temperature. Analyses and visualizations were conducted in R 4.4.1 (R Core Team 2024).

## 2 Results

### 2.1 Soil Temperature and Soil Water Content

**FIGURE 3:**
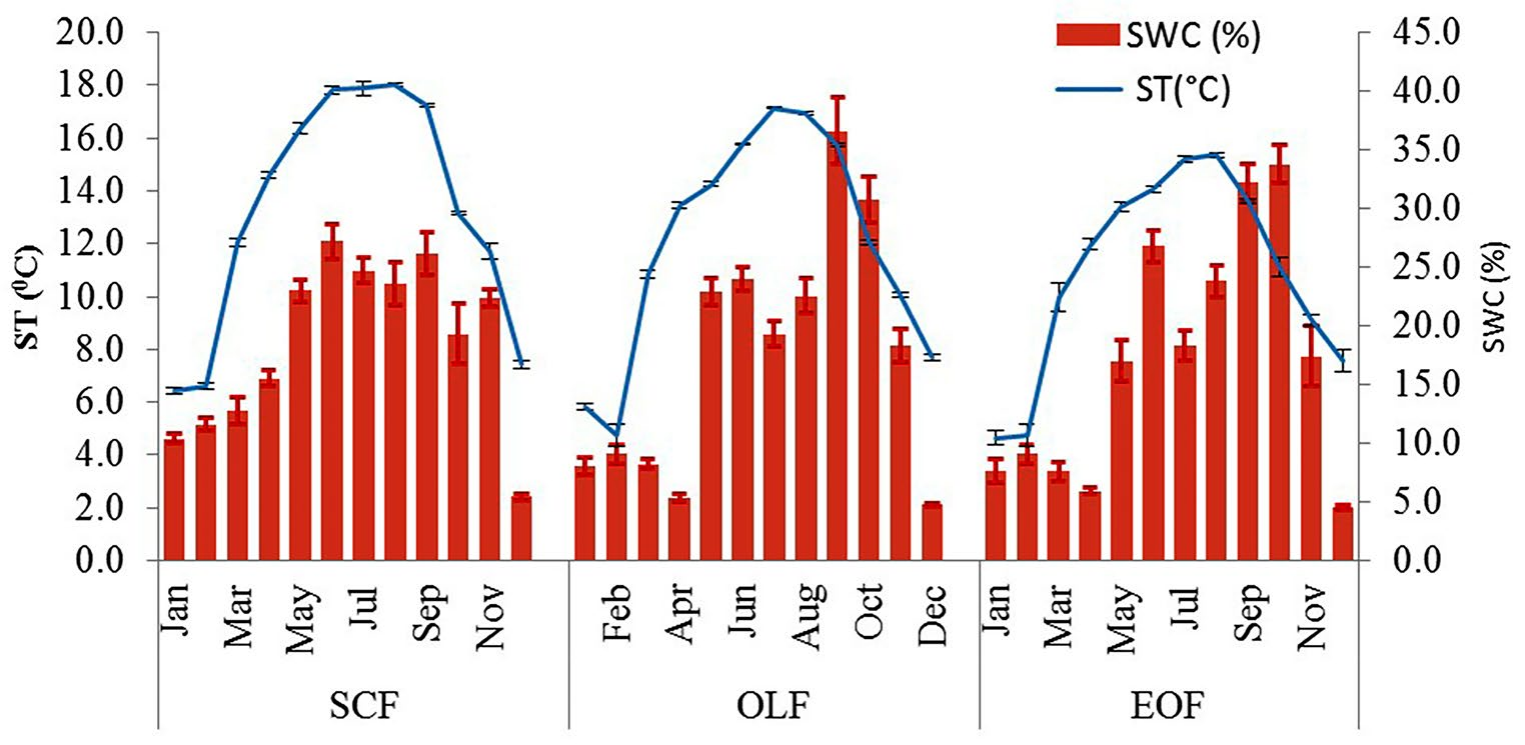
Monthly variation of soil temperature (°C) and soil water content (%) in forests. EOF: Evergreen Oak Forest; OLF: Oak‐Laurel forest; SCF: *Schima‐Castanopsis* Forest.

Soil temperature and soil water content varied significantly (*p* < 0.001) among the forest types. Seasonal changes in soil temperature showed a similar tendency in all the forest types with a peak value during summer. Soil moisture content was also found variable among the seasons. The average soil temperature was 13.3°C, 12°C, and 10.9°C in the studied forests: *Schima‐Castanopsis*, Oak‐Laurel and Evergreen Oak forest, respectively. The minimum soil temperature (4.5°C) was measured in Evergreen Oak Forest during December, whereas the maximum value was recorded in *Schima‐Castanopsis* Forest (18°C) during August. Soil water content was irregular depending on season and forest sites with lowest measure during December (Figure 3). The highest soil water content was recorded in June in the *Schima‐Castanopsis* forest whereas in Oak‐Laurel forest, peak values were obtained during September and October. In the *Schima‐Castanopsis* Forest, soil water content (SWC) showed a clear seasonal pattern. It was relatively low in winter (January–March), and then gradually increased from spring to early summer, reaching a peak during the monsoon period (around June–September).

### 2.2 Soil CO_2_ Efflux in Different Forest Types

**FIGURE 4:**
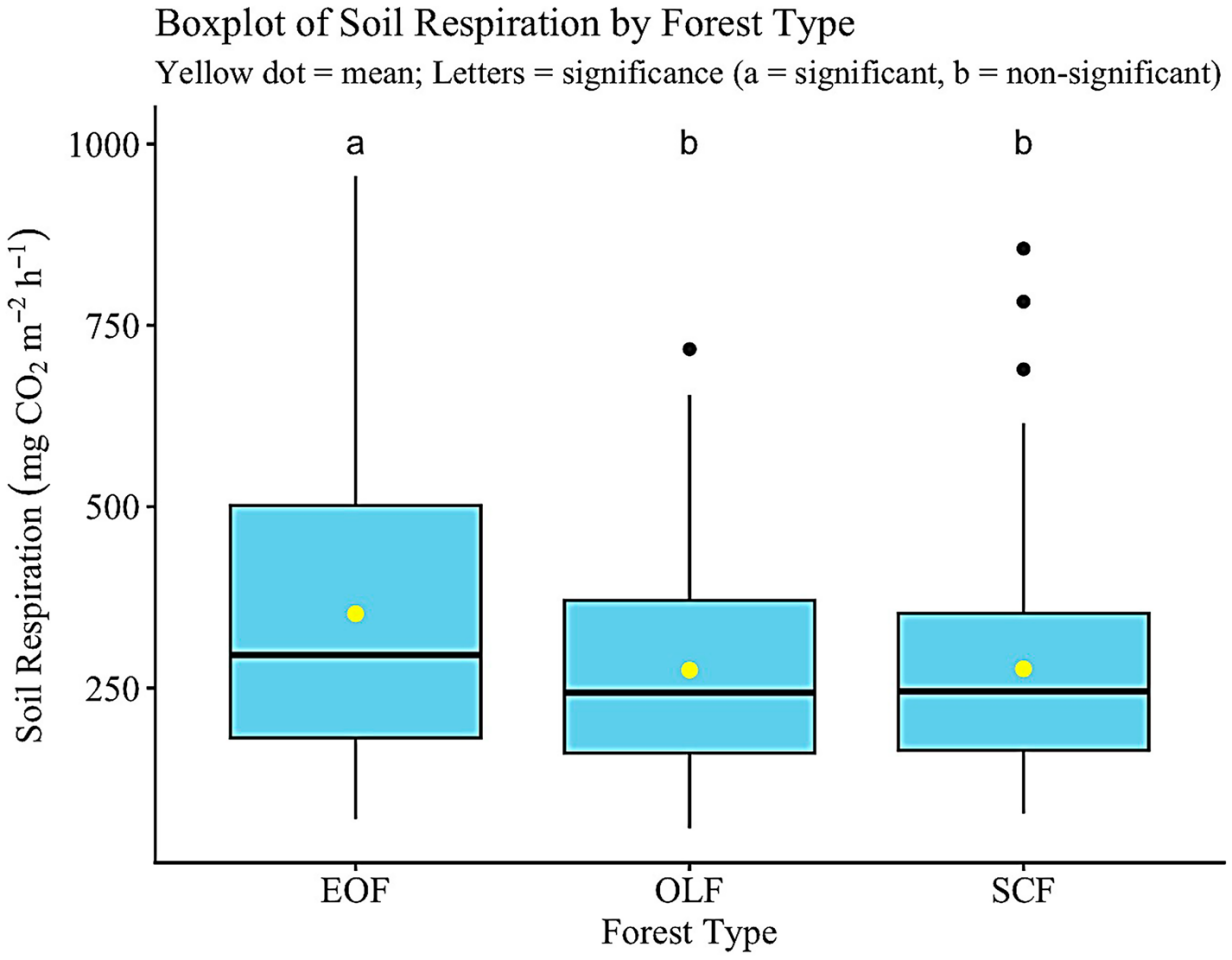
Soil respiration rate in the three forest types (EOF: Evergreen Oak Forest; OLF: Oak‐Laurel forest; SCF: *Schima‐Castanopsis* Forest); yellow dot = mean; letters = significance, a = significant, b = nonsignificant.

**FIGURE 5:**
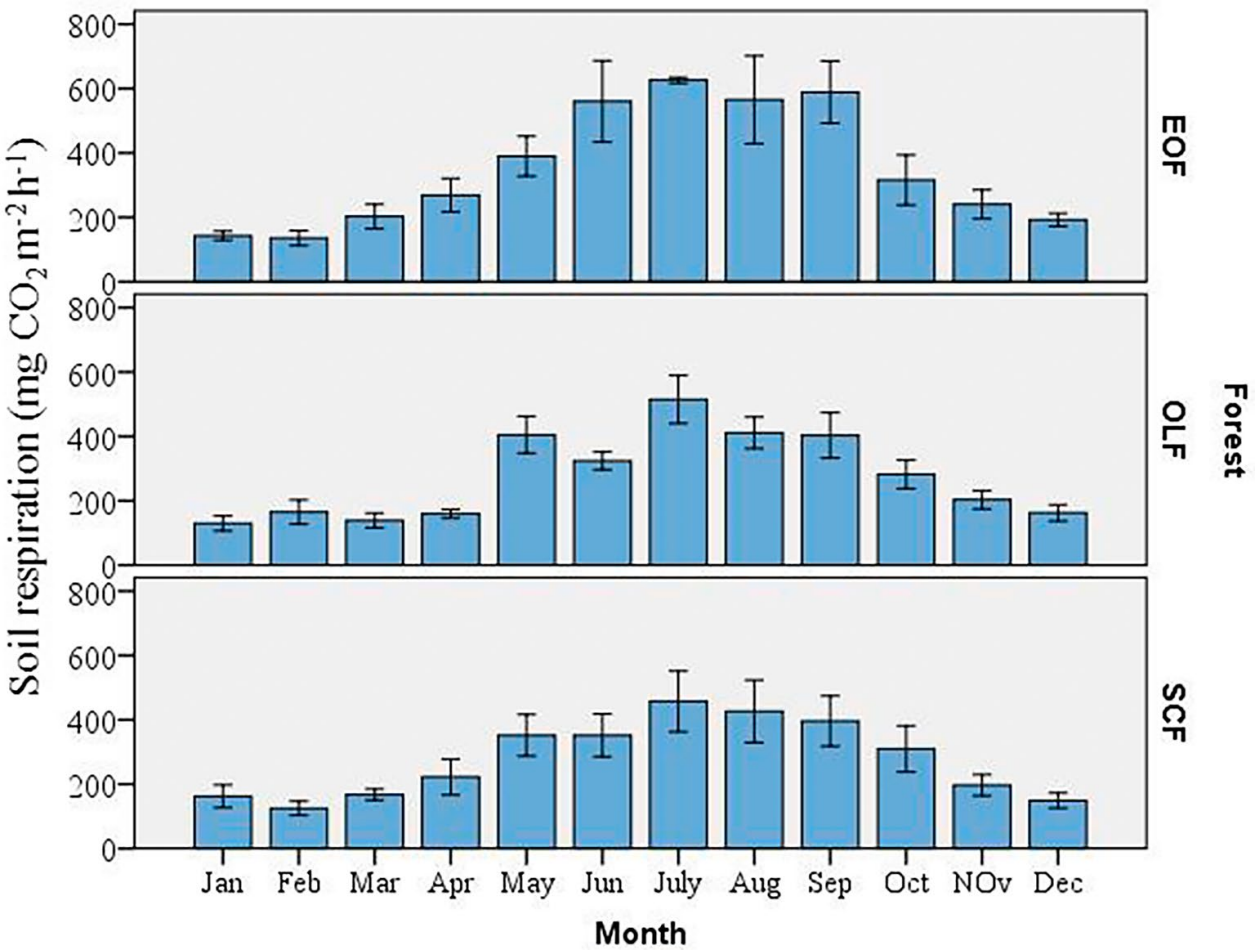
Monthly variation of soil respiration rate (mg CO_2_ m^−2^ h^−1^). EOF: Evergreen Oak Forest; OLF: Oak Laurel forest; SCF: *Schima‐Castanopsis* Forest.

Soil respiration rate varied significantly (*p* = 0.002) among the three forest types. The mean soil CO_2_ efflux rates were 352.4, 274.7, and 276.3 mg CO_2_ m^−2^ h^−1^ in the three forests Evergreen Oak Forest (EOF), Oak‐Laurel Forest (OLF), and *Schima‐Castanopsis* Forest (SCF), respectively (Figure 4). The highest soil respiration rate was recorded in Evergreen Oak Forest (upper elevation), while the lowest was observed in Oak–Laurel Forest (middle elevation). Year‐round measurements showed a clear seasonal pattern, with soil respiration peaking in July and reaching its lowest values during January–February. This trend was consistent across all three forest types (Figure 5). In *Schima‐Castanopsis* Forest, soil respiration ranged from 125 to 457.2 mg CO_2_ m^−2^ h^−1^. The minimum value was recorded in February, followed by a steady increase until July, after which it gradually declined (Figure 4). Similarly, in Oak‐Laurel Forest, soil respiration rate ranged from 129.5 mg CO_2_ m^−2^ h^−1^ in January to a maximum of 514.5 mg CO_2_ m^−2^ h^−1^ in July (Figure 5). A comparable pattern was observed in Evergreen Oak Forest, with the lowest respiration in February (135.1 mg CO_2_ m^−2^ h^−1^) and the highest in July (325.9 mg CO_2_ m^−2^ h^−1^).

**FIGURE 6:**
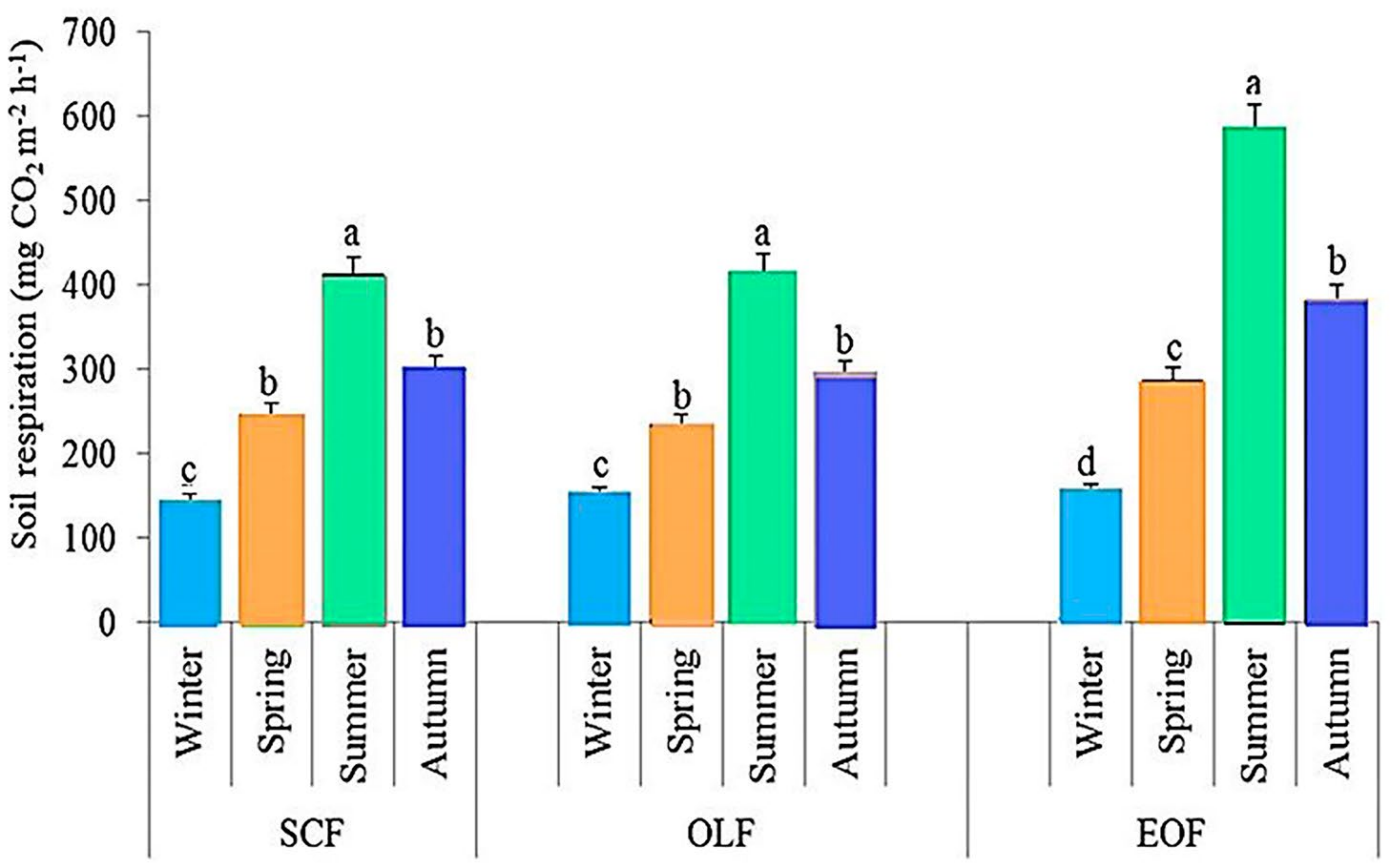
Seasonal variation of soil respiration rate in the three forest types; the alphabets ‘a–d’ indicates significant differences among the seasons in each forest type. EOF: Evergreen Oak Forest; OLF: Oak Laurel forest; SCF: *Schima‐Castanopsis* Forest.

The seasonal dynamics of soil respiration also exhibited a pronounced peak, with the maximum values recorded in summer. Soil respiration varied significantly (*p* < 0.001) across seasons in all the forest types, with mean values following the order: summer > autumn ≥ spring > winter (Figure 6). Soil respiration rates were lowest during winter and reached their peak during summer (monsoon season) in all the forest sites. The highest soil respiration rate was measured in summer, that is, 583.8 mg CO_2_ m^−2^ h^−1^ in Evergreen Oak Forest whereas the lowest rate was observed during the winter season at *Schima‐Castanopsis* Forest (145.6 mg CO_2_ m^−2^ h^−1^).

**FIGURE 7:**
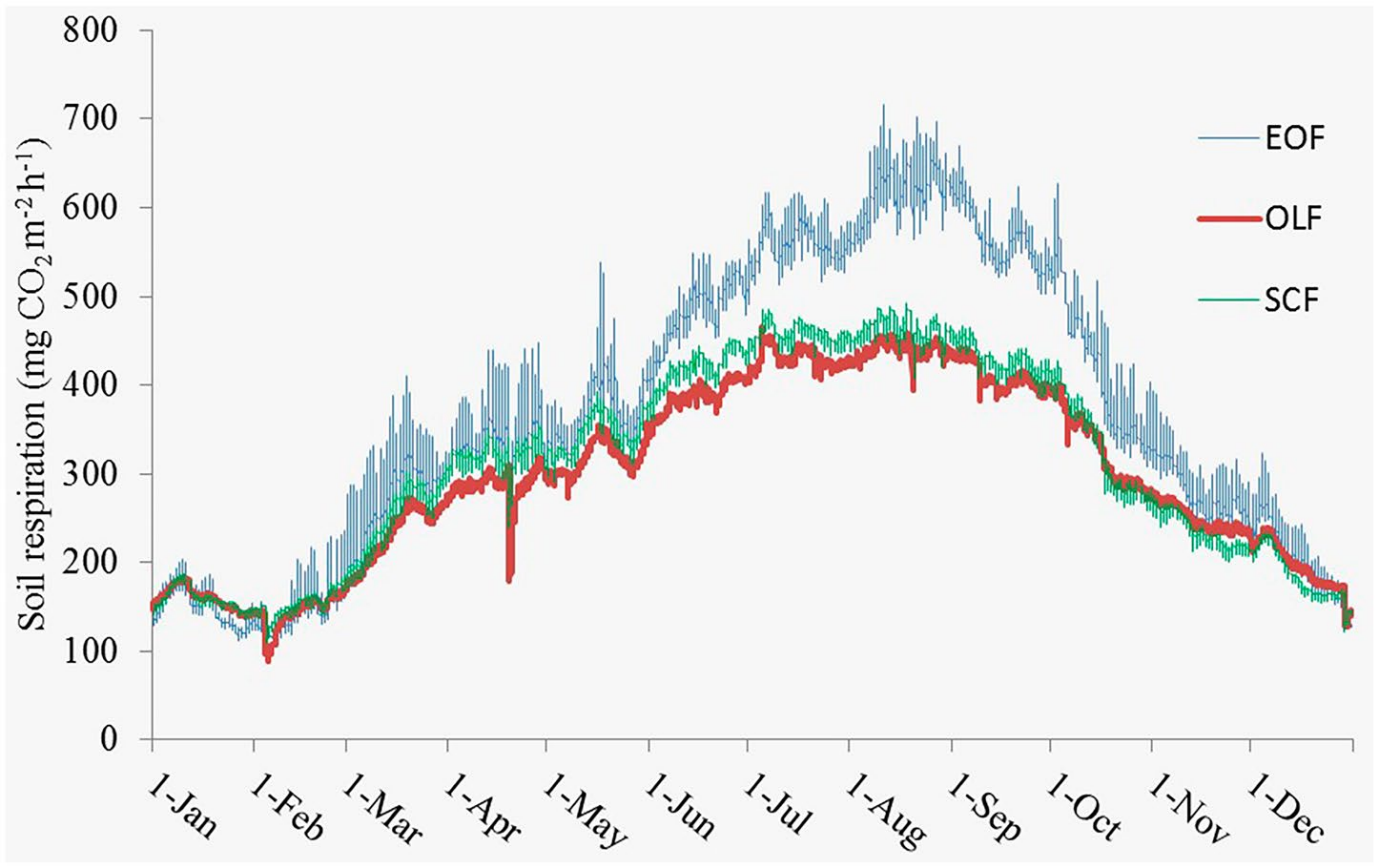
Soil respiration (hourly) from January to December in the three forests (EOF: Evergreen Oak Forest; OLF: Oak Laurel forest; SCF: *Schima‐Castanopsis* Forest).

The estimated annual soil CO_2_ efflux for the measurement period January to December ranged from 2614.9 to 3260.4 g CO_2_ m^−2^ year^−1^. Since CO_2_ efflux measurements were conducted during daytime only, annual soil respiration was modeled using continuous soil temperature data recorded by a TidbiT data logger (HOBO MX2204). Consistent with the hourly soil respiration rates, annual soil respiration was highest in the Evergreen Oak Forest at the upper elevation belt (Figure 7). Annual carbon emission from soil respiration ranged from 706 to 880.3 g C m^−2^ year^−1^ in the three forest types (Table 2).

**TABLE 2:** Annual soil respiration and temperature sensitivity to soil respiration (Q10) values in the three forest types.

Elevation belt	Forest type	Q10 value	Soil respiration (mg CO_2_ m^−2^ h^−1^)	Annual respiration (g CO_2_ m^−2^ year^−1^)	Annual soil carbon efflux (g C m^−2^ year^−1^)
Upper	EOF	3.9	352.4	3260.4	880.3
Middle	OLF	2.7	274.7	2614.9	706.0
Lower	SCF	2.5	276.3	2717.8	733.8

### 2.3 Response of Soil Respiration to Environmental Variables

#### 2.3.1 Response of Soil Respiration to Soil Temperature

**FIGURE 8:**
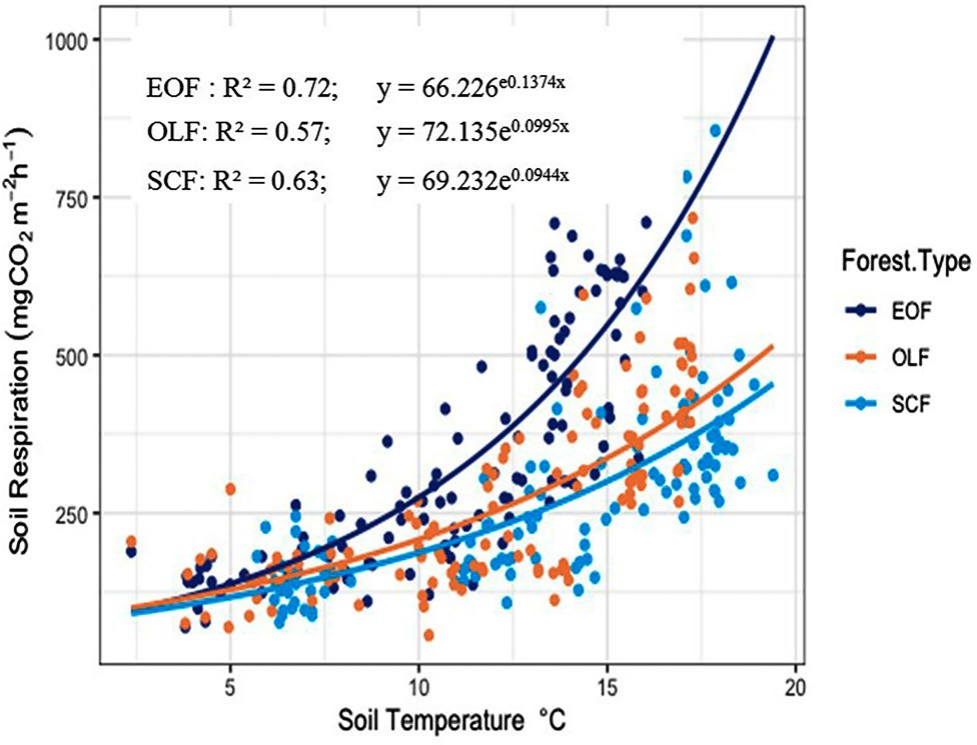
Relation between soil respiration and soil temperature (the dark blue circle, brown circle, and light blue circles indicate the points of data in EOF (Evergreen Oak Forest), OLF (Oak Laurel forest), and SCF (*Schima‐ Castanopsis* Forest), respectively); the *R* ^2^ values are pseudo *R* ^2^ derived from nonlinear model and therefore represent an approximate measure of model fit.

Soil temperature is an important factor influencing soil respiration. The year round measurement showed that there was a significant positive relationship between soil respiration rate and soil temperature (*p* < 0.001). The soil temperature accounted for 72%, 57%, and 63% of the variation in soil respiration rate in the three forests Evergreen Oak, Oak‐Laurel, and *Schima‐Castanopsis* Forest, respectively (Figure 8).

The temperature sensitivity of soil respiration (Q10) increased from 2.5 at 1600 m (*Schima‐Castanopsis* Forest) to 2.7 at 2000 m (Oak‐Laurel Forest) and 3.9 at 2600 m (Evergreen Oak Forest), respectively. Hence highest Q10 value was found in Evergreen Oak Forest at higher elevation. Temperature sensitivity (Ql0) of soil respiration indicates the factor by which the soil respiration rate increases with an increment of soil temperature by 10°C.

#### 2.3.2 Response of Soil Respiration to Soil Water Content

**FIGURE 9:**
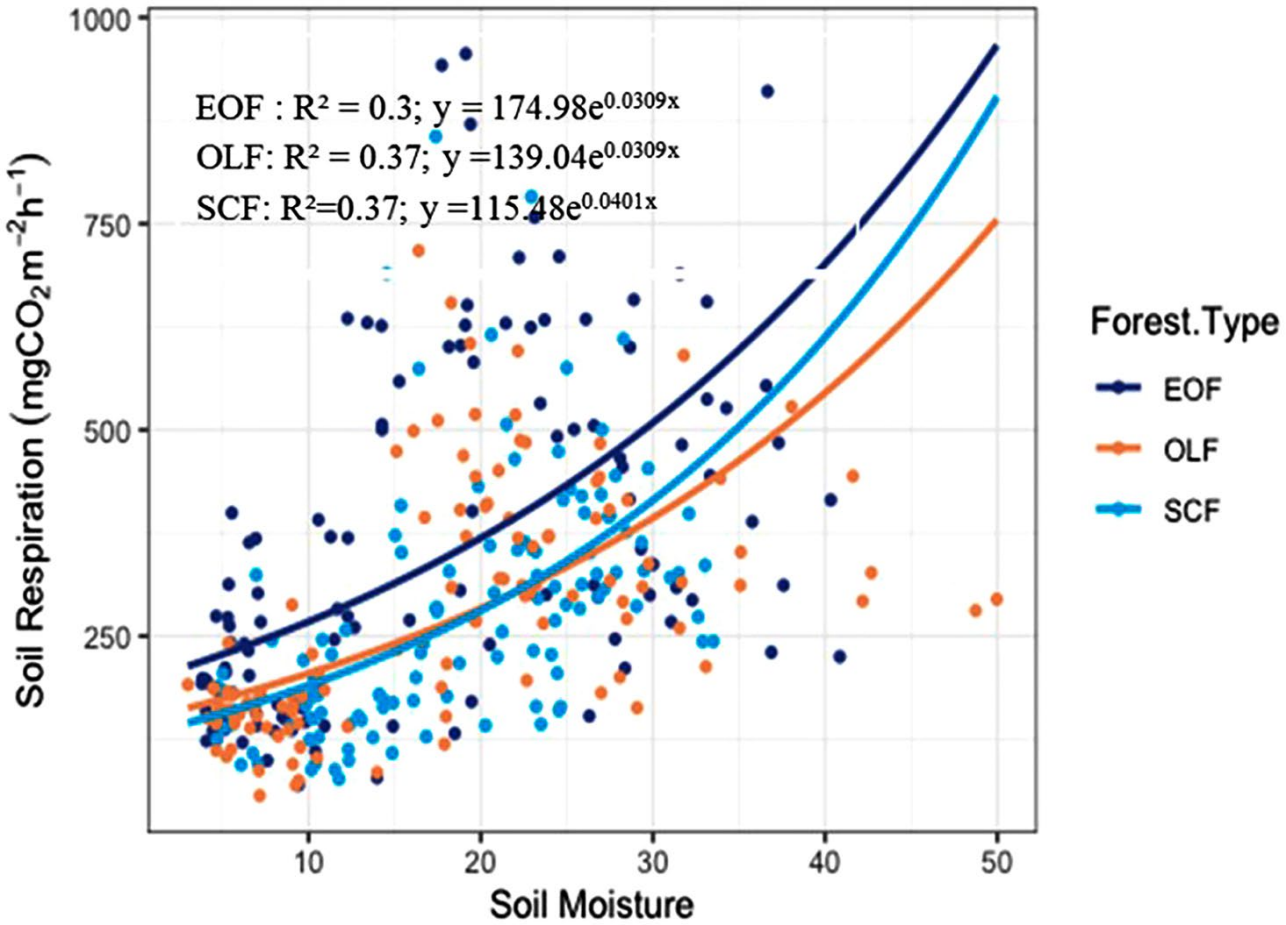
Relation between soil respiration and soil moisture (The dark blue circle, brown circle, and light blue circles indicate the points of data in EOF (Evergreen Oak Forest), OLF (Oak‐Laurel forest), and SCF (*Schima‐Castanopsis* Forest), respectively).

There was a significant exponential relationship (*p* < 0.001) between soil respiration and soil water content in forests across all altitudes. Soil respiration showed a significant but weaker relationship with soil water content than soil temperature. Soil water content accounted for 30%–37% of the soil respiration variation among the three forest types (Figure 9).

## 3 Discussion

The study was conducted across three broadleaved forest types with varying tree species compositions and elevation ranges. Soil temperature and moisture content varied with seasons and forest types. This trend is mainly driven by changes in air temperature and solar radiation with seasons. Higher elevation forests (like Evergreen Oak Forest) generally showed lower average soil temperatures due to cooler climatic conditions. In *Schima‐Castanopsis* Forest, soil moisture seems strongly controlled by seasonal precipitation, particularly the monsoon. The difference in soil moisture (SWC) during September–October across the three forests mainly reflects the difference in precipitation in varying altitudes and site‐specific factors such as canopy structure, litter layer, and soil characteristics (Haifang et al. 2019).

Significant differences in soil respiration rates, ranging from 274.7 to 372.4 mg CO_2_ m^−2^ h^−1^, were observed among the three forests along elevation gradient. Wide variations in soil CO_2_ emission rates have been reported in various forest types worldwide. For example, the rate of soil respiration in the oak forest of Kumaun Himalaya, India, ranged from 63.9 to 363.6 mg CO_2_ m^−2^ h^−1^ (Jina et al. 2008). In Nepal, soil respiration measurements in different forest types varied from 270 to 295 mg CO_2_ m^−2^ h^−1^ in *Rhododendron* forests (Dhital, Manandhar, Gosain, and Sijapati 2022), 148 to 892 mg CO_2_ m^−2^ h^−1^ in subtropical broadleaved forests (Dhital, Manandhar, Manandhar, and Maharjan 2022) and 311.8 mg CO_2_ m^−2^ h^−1^ in pine forests (Dhital, Gosain, and Maharjan 2022). Our findings estimate annual soil respiration rates ranging from 706 to 880 g C m^−2^ year^−1^. Annual soil respiration rates varied from 203 to 1290 g Cm^−2^ year^−1^, with a mean value of 669 g C m^−2^ year^−1^ in forests of Japan (Lee et al. 2006). Thus, our findings on soil respiration fall within the estimated ranges reported for other forests. Soil respiration rates differ between forest types in different climatic zones due to variation in temperature and rainfall patterns. Further, the annual soil respiration rate in our study was lower than the reported values of 2560 g C m^−2^ year^−1^ by Hashimoto et al. (2004) in tropical monsoon forests and 1300 to 1567 g C m^−2^ year^−1^ by Jiang et al. (2017). Lower respiration rates observed in this study are likely attributable to climatic differences between subtropical/temperate forests and tropical monsoon forests. This study limits soil respiration measurement for 1 year period. Long‐term studies on soil respiration are essential to analyze inter‐annual variation of carbon efflux in these ecosystems.

There is general consensus that soil respiration rate decreases with elevation due to a decline in temperature (Badraghi et al. 2021) and lower plant biomass (Takeda et al. 2023). In contrast, our findings showed the highest soil respiration rate in the Evergreen Oak Forest of the upper elevation belt. The variation of soil carbon emissions across different forests may be attributed to differences in vegetation structure, micro‐environmental conditions, and substrate structure (Raich and Tufekciogul 2000). Trees of the Evergreen Oak Forest exhibit a mature stage with predominantly large‐sized trees. Vegetation types influence soil respiration by affecting the physical environment, nutrient availability, and carbon dynamics (Raich and Tufekciogul 2000). Soil respiration has been shown to be affected by changes in vegetation structure than by changes in tree species. Along with climatic factors, vegetation structure (density and abundance) and topographic conditions are also major determinants of soil respiration variation across different sites (Pandey et al. 2023). Soil respiration is positively correlated with root biomass (Lee 2018) and litter input (Zhuang et al. 2023). Hence, higher soil respiration in the Evergreen Oak Forest at upper elevation could be due to higher tree/root biomass, litter input, and older stand age (Wu et al. 2020). This contradicting result could also be due to enhanced root activity during favorable seasons (summer/growing season), primarily due to enhanced autotrophic (root) respiration (Kumar et al. 2023).

Soil respiration rate showed a significant exponential relationship (*R* ^2^ ranging from 57% to 72%) with the soil temperature in forests of different elevation. Numerous studies demonstrate that soil respiration responds exponentially to soil temperature in different forest ecosystems (Dhital, Manandhar, Gosain, and Sijapati 2022; Wang et al. 2024; Yan et al. 2024). Soil respiration rate increases with soil temperature due to changes in biological and biochemical activities within the soil (Borowik and Wyszkowska 2016; Qu et al. 2023). Soil temperature has influence on plant growth, activity of microorganisms and substrate supply (Hanson et al. 2000). The seasonal variation in soil respiration reflects the changes in soil temperature and moisture conditions. Also, seasonal fluctuations in temperature regulate the decomposition of organic matter, thereby controlling the activity and respiration by soil microorganisms (Pan et al. 2026). On the other hand, the relationship signifies that higher temperature results in increased rates of carbon emission (Davidson et al. 2006).

Along with temperature, soil moisture is also an important driver of temporal variation in soil respiration rate. Seasonal fluxes of soil respiration correlate with soil moisture along with temperature (Yan et al. 2024). However, soil respiration rate may be followed by a decrease with temperature after a threshold value (Wang et al. 2019). Studies suggest that soil respiration owes to the interacting effect of both temperature and moisture. In our study, soil respiration showed a relatively weak correlation with soil moisture than with soil temperature. Soil respiration rates have a positive correlation with soil moisture to a certain limit of 23%–41% (Lee et al. 2006). The rate may show a negative relationship either in higher soil moisture due to poor aeration or due to desiccation in too low soil water content (Janssens and Pilegaard 2003). Soil moisture increases productivity and carbon inputs (Moyano et al. 2013). Soil respiration in Nepalese forests shows seasonal variation, primarily driven by changes in soil moisture and rainfall patterns associated with the monsoon climate. During the dry season (March–May), soil moisture was lower than in summer and autumn due to limited rainfall and higher temperature. Lower soil moisture restricts microbial activity and root respiration even though temperatures are favorable (Lima et al. 2023). Soil respiration is often affected by the interaction of multiple factors, although separating their interactions is quite challenging.

Temperature sensitivity (Q10) value represents a coefficient that indicates temperature‐dependence of soil respiration. The Q10 value of soil respiration ranged from 2.5 to 3.95 in the studied forests, indicating a gradual increase with elevation. A wide range of Q10 values has been documented; for instance, 3.06–4.49 in forests of China (Ma et al. 2019), a mean value of 2.46 (Song et al. 2014), and 1.4–4.6 in tropical and subtropical forests (Chen and Tian 2005). The obtained Q10 value falls within the range reported for Himalayan ecosystems 0.47 to 4.97 (Tiwari et al. 2021) and 1.4 to 6.2 (Dhital et al. 2019). Q10 values typically show large variability with ecosystem types, and seasonal change due to strong temperature–moisture interactions. Compared to global averages 2–3 (Raich and Schlesinger 1992), our estimates often exhibited higher and more variable Q10 values, reflecting greater sensitivity of soil respiration to temperature in cold environments.

Temperature sensitivity (Q10) of soil respiration increased with elevation in forests of Australia with Q10 = 2.63 at highest elevation (Zimmermann et al. 2015). There is consensus that Q10 increases with latitude from tropical to temperate and further to Polar Regions (Bekku et al. 2004). The Q10 value of soil respiration increases with elevation due to more soil carbon storage but lower decomposition in cold climate. Higher Q10 value in Evergreen Oak Forest, at upper elevation suggests that soil carbon storage in this forest may suffer more disturbances with much soil carbon emission under global warming. Hence, temperate Evergreen Oak forests exhibit higher soil respiration rate despite their distribution at upper elevations. Himalayan ecosystems are warming at roughly three times the global average, leading to significant changes in vegetation and soil–atmosphere CO_2_ exchange (Kumar and Khanduri 2024). In Nepal, a recent study on climate change impacts revealed projections of temperature increase by 4°C–6°C and an increase in precipitation by 40%–60% by the end of the 21st century (Dhital et al. 2023). With rising temperatures in the future, these forests are likely to release more carbon from soil due to increased root respiration and the enhanced activity of microorganisms adapted to cooler climates.

## 4 Conclusion

Our study highlights the variation of soil respiration rate in the forests along the elevation gradient of Nepal. The highest soil respiration rate was recorded in the Evergreen Oak Forest at upper elevation. A significant exponential relationship of soil respiration with the soil temperature and the soil water content was observed in all the three forests. The seasonal variation in soil temperature and moisture accounts for seasonal changes of soil respiration. Temperature sensitivity to soil respiration (Q10) increased in forests along the elevation gradient, indicating the trend of enhanced efflux of soil CO_**2**_ from forests at higher elevation in the context of future climate warming. This study underscores the importance of incorporating belowground carbon processes into climate policy frameworks and sustainable forest management strategies in Nepal. Long‐term studies are particularly necessary in high‐altitude forests, which are more sensitive to climate change.

## Funding

The study was funded by Nepal climate Change and Knowledge management Center (NCCKMC), Nepal Academy of Science and Technology (NAST), Nepal. The funder had no role in study design, data collection and analysis, decision to publish, or preparation of the manuscript.

## Ethics Statement

The study complied with institutional and national ethical guidelines. No external entity influenced the study design, data collection, analysis, interpretation of results, or the decision to submit the work for publication. All authors have reviewed and approved this statement.

## Conflicts of Interest

The authors declare no conflicts of interest.

## Supporting information


**Data S1:** Supporting information.

## Data Availability

The data that supports the findings of this study are available in the Supporting Information S1 of this article.
